# Outcome of Successful Versus Unsuccessful Percutaneous Coronary Intervention in Chronic Total Occlusions in One Year Follow-Up

**DOI:** 10.4021/cr258w

**Published:** 2013-05-09

**Authors:** Bahram Sohrabi, Samad Ghaffari, Afshin Habibzadeh, Parastoo Chaichi, Amir Kamalifar

**Affiliations:** a Dept. of Cardiology, Cardiovascular Research Center, Tabriz University of Medical Sciences, Tabriz, Iran; b Medical Philosophy and History Research Center, Tabriz University of Medical Sciences, Tabriz, Iran; c Students’ Research Committee, Tabriz University of Medical Sciences, Tabriz, Iran

**Keywords:** Chronic total occlusion, Percutaneous coronary intervention, Coronary artery disease, Outcome, Follow-up

## Abstract

**Background:**

Chronic total occlusions (CTO) comprises already one-third of percutaneous coronary interventions (PCIs). There is controversy in PCI results considering short-term and long-term outcomes. We aim to compare efficacy and outcome of successful versus unsuccessful PCI in CTO in 1 year follow-up.

**Methods:**

In this retrospective study we choose 330 consecutive patients undergone PCI on a CTO of a native coronary artery (163 successful and 167 unsuccessful) in Madani Heart Hospital, Tabriz, Iran. Patients were followed for a mean period of about 15 ± 3 months. Major adverse cardiac events (MACE) in hospital and in follow-up were recorded comprising death, acute myocardial infarction, and need for repeat revascularization.

**Results:**

Patients with unsuccessful PCI compared to successful PCI were mainly male (87.4% vs. 77.3%; P < 0.02), had a higher incidence of diabetes mellitus (31.1% vs. 20.9%; P < 0.04) and hypertension (53.3% vs. 42.3%; P < 0.04). Most patients in successful group had single vessel disease (63.4% vs. 46.7%; P < 0.001) and less three-vessel disease (11.8% vs. 22.8%) compared to unsuccessful group. In-hospital MACE was insignificantly higher in unsuccessful PCI (17.4% vs. 11%). Unsuccessful PCI was significantly associated with higher rate of 12 months MACE (43.7% vs. 30.1%, P = 0.01), especially revascularization (41.3% vs. 25.2%, P = 0.02).

**Conclusion:**

Although in hospital outcome was the same between groups, patients with successful PCI of CTO had a better one year follow-up outcome than unsuccessful PCI. However mortality rate was the same and main complications were due to revascularization.

## Introduction

Approximately one-third to one-half of patients with significant coronary artery disease on angiography has at least 1 CTO [1-4]. During the past 25 years the use of percutaneous coronary intervention (PCI) has become common in the management strategy of patients with chronic coronary total occlusion (CTO) [5]. However CTO is a very common reason not to attempt percutaneous coronary intervention (PCI) [1], as it has a lower success rate than for those of other de novo coronary lesions [6].

Because of low procedural success rate [5, 7], high restenosis rate [8], and high incidence of adverse events [6, 9] documented in the early reports of PCI, a large population of patients with CTOs have been managed medically, and such lesions have been the most common reason for referral to bypass surgery rather than PCI [10].

Patients with successful recanalization of CTO with PCI have better symptom relief, better clinical outcome, improved left ventricular function and better long-term survival compared with patients in whom the attempt to re-canalize CTO has failed [6, 7, 9, 11-16]. The aim of current study is to compare efficacy and outcome of successful versus unsuccessful percutaneous coronary intervention in chronic total occlusions. It’s the first report in our area that compares midterm results of these patients.

## Methods and Materials

### Study design

This single center, retrospective, observational clinical study was conducted in Madani Heart Center, Tabriz, Iran. For this purpose, 163 cases with successful PCI and 167 cases with unsuccessful PCI were enrolled in the study. The study protocol was approved by the institutional ethics review board. All procedures were followed in accordance with the Declaration of Helsinki.

Patients requiring non-emergency PCI to treat a 100% occlusion of a coronary artery were included. The exclusion criteria were the estimated duration of a CTO < 30 days or an acute myocardial infarction (MI) within the previous 30 days. No other predefined clinical inclusion or exclusion criteria were considered, and the indication for PCI was decided by individual investigators at the participating center.

### Definitions

CTO was defined as a lesion showing thrombolysis in myocardial infarction grade of 0 of 3 months or more in duration. All patients included in this analysis have at least 1 occlusive lesion. Duration of occlusion was estimated on the basis of history of angina, previous myocardial infarction (MI) in the same territory, or proven by previous angiography.

Major adverse cardiac events (MACE) were defined as death, Q-wave MI, non-Q-wave MI, or urgent revascularization during the same admission. Q-wave MI was defined as cardiac enzyme (creatine kinase) elevation of > 3 times the normal value with concomitant elevation of creatine kinase myocardial-band > 3 times and development of Q wave after the PCI. Non-Q-wave MI was defined as elevation of creatine kinase > 3 times without development of Q wave after the PCI.

Procedural success was defined as successful guide wire and balloon crossing with residual stenosis > 50% and Thrombolysis in Myocardial Infarction grade 3 flow.

## Methods

All patients were pre-treated with aspirin and clopidogrel (a loading dose of 300 mg at least 6 h before the procedure). After the procedure, all patients were given aspirin indefinitely and clopidogrel 75 mg daily at least 3 or 6 months after implantation of drug eluting stents. Glycoprotein IIb/IIIa inhibitors were given at the discretion of the operator.

Information was retrospectively recorded, including baseline demographics, clinical and procedural characteristics, and in-hospital outcomes. Patients were followed for 1 year and MACE in hospital and in one year follow-up were recorded.

Angiographic success was defined as successful balloon dilatation of the lesion, with or without stent placement, with less than 40% residual stenosis. Procedural success was defined as angiographic success with no in-hospital MACE, defined as death, MI with new Q-waves on electrocardiogram (ECG) or urgent target vessel revascularization (TVR) (including both repeat PCI and coronary artery bypass graft surgery (CABG)). New MI was defined as elevation of creatine kinase-MB to > 2 times the upper limit of normal with recurrent ischemic symptoms following PCI. Post-procedural ECGs were routinely assessed for new Q-waves; however, cardiac troponin, creatine kinase and creatine kinase-MB fraction were not routinely collected.

Follow-up protocol included evaluation at hospital discharge and a clinical visit or telephone interview after discharge and at 3, 6 and 12 months. Patients that developed angina or objective evidence of target vessel ischemia in follow up period underwent angiography.

### Data analysis

Continuous data with normal distribution are given as mean ± standard deviation, otherwise as median, student t test for testing the significance of mean for independent continuous scale data and Mann-Whitney U test for nonparametric data where appropriate, Chi-square or Fisher exact test for testing the significance of percentages. A p value of 0.05 or less was considered significant.

## Results

### Baseline characteristics of the study population

We enrolled 330 patients (163 successful and 167 unsuccessful PCI) with at least 1 CTO lesion in the combined registry. Patients with unsuccessful PCI were mainly male (87.4% vs. 77.3%; P < 0.02), had a higher incidence of diabetes mellitus (31.1% vs. 20.9%; P < 0.04) and hypertension (53.3% vs. 42.3%; P < 0.04) when compared to patients with successful CTO revascularization (Table 1). A history of previous MI was present in nearly half of the patients in both groups.

**Table 1:** Baseline Clinical Characteristics in the Entire Cohort of Patients

Variable	CTO success (n = 163)	CTO failure (n = 167)	P value
Age, years	58.20 ± 11.06	59.22 ± 11.26	NS
Male, sex	126 (77.3%)	146 (87.4%)	0.02
Diabetes mellitus	34 (20.9%)	52 (31.1%)	0.04
Hyperlipidemia	54 (31.3%)	59 (35.3%)	NS
Hypertension	69 (42.3%)	89 (53.3%)	0.04
Smoking current	54 (31.3%)	58 (34.7%)	NS
Familial history	24 (14.8%)	33 (19.8%0	NS
Previous Myocardial infarction	68 (41.7%)	78 (47.3%)	NS
Previous PCI	60 (36.8%)	65 (38.9%)	NS
Previous CABG	13 (8%)	19 (11.4%)	NS
Renal insufficiency	2 (1.2%)	4 (2.4%)	NS
Cerebrovascular disease and arrest	1 (0.6%)	2 (1.2%)	NS
Peripheral vascular disease	36 (22.1%)	26 (15.6%)	NS
LVEF < 40%	41 (25.2%)	51 (30.5%)	NS

CTO: Coronary total occlusion; PCI: Percutaneous coronary intervention; CABG: Coronary artery bypass graft; LVEF: Left ventricular ejection fraction; NS: not significant.

Table 2 summarizes procedural characteristics according to success and failure groups. Most patients in success group had single vessel disease (63.4% vs. 46.7%; P < 0.001) and less three-vessel disease (11.8% vs. 22.8%) when compared to patients with unsuccessful CTO revascularization.

**Table 2:** Procedural Characteristics According to CTO Success and Failure Groups

Variable	CTO success (n = 163)	CTO failure (n = 167)	P value
Target vessel of intervention, No. (%)			< 0.001
Left anterior descending	98 (60.1%)	60 (35.9%)	
Circumflex	26 (16%)	29 (17.4%)	
Right coronary artery	39 (23.9%)	78 (46.7%)	
Number of diseased vessels, No. (%)			0.005
1	102 (63.4%)	78 (46.7%)	
2	40 (24.8%)	51 (30.5%)	
3	19 (11.8%)	38 (22.8%)	

CTO: Coronary total occlusion.

Procedural complications observed in 4 patients (all male) including tamponade while PCI of CTO. All patients had failed PCI. CTO was on LAD (2 cases) and RCA (2 cases). Three had 2 vessels and 1 had 3 vessels disease.

Success rate was significantly higher in LAD (62%) than Lcx (47.3%) and RCA 33.3% (P < 0.001). Also among different vessel involvement, one vessel disease (56.7%) had significantly higher success rate than multivessel disease (2 vessel (44%) and 3 vessel (33.3%)) (P = 0.005).

### In-hospital and one year clinical outcomes

A summary of clinical outcomes is shown in Table 3. In-hospital MACE was insignificantly higher in patients with failed PCI compared to successful PCI (17.4% vs. 11%). Unsuccessful PCI was associated with a significantly higher rate of 12 months MACE (43.7% vs. 30.1%, P = 0.01), especially revascularization (41.3% vs. 25.2%, P = 0.02).

**Table 3:** In-Hospital and 12-Months Follow-Up Events in Patients With CTO

Variable	CTO success (n = 163)	CTO failure (n = 167)	P value
In-hospital MACE, N (%)	18 (11%)	29 (17.4%)	NS
Cardiac death	1 (0.6%)	2 (1.2%)	NS
Myocardial infarction	2 (1.2%)	4 (2.4%)	NS
Revascularization	16 (9.8%)	23 (13.8%)	NS
Target Vessel	11 (68.8%)	11 (47.8%)	
Other vessel	5 (31.2%)	12 (52.2%)	
Bleeding	2 (1.2%)	6 (3.6%)	NS
MACE in 12 months	49 (30.1%)	73 (43.7%)	0.01
Cardiac death	2 (1.2%)	0	NS
Non-cardiac death	3 (1.8%)	3 (1.8%)	NS
Myocardial infarction	12 (7.4%)	12 (7.2%)	NS
Revascularization	41 (25.2%)	69 (41.3%)	0.002
Target Vessel	26 (63.4%)	41 (59.4%)	
Other vessel	15 (36.6%)	28 (40.6%)	

CTO: Coronary total occlusion; MACE: Major adverse cardiac evets; NS: not significant.

## Discussion

In this study, we compared clinical and one year follow-up outcome in 163 successful and 167 unsuccessful PCI on CTO. Patients with unsuccessful PCI were mainly male, had a higher incidence of diabetes mellitus and hypertension. Clinical outcome was improved significantly if the recanalization for CTO was successful, reflected by the lower rate of MACE within the twelve months follow-up period. Notably, no death or new MI was observed in the hospital after the procedures. Moreover, the need for the repeat revascularization of successfully opened occlusions was acceptably low. The risk for late death or myocardial infarction was similar whether or not a patient was discharged with an open artery during the mean follow-up period of 12 months.

Together with the treatment of diffuse multivessel disease and in-stent restenosis lesions, treatment of CTO lesions is considered as one of the remaining major challenges facing interventional cardiologists and the treatment strategy of multivessel disease is currently affected by the presence of CTOs. According to a report [14], 73% of patients without a CTO are referred to PCI, but in the presence of CTO only 47% are referred.

In our study success rate was significantly higher in LAD (62%) than Lcx (47.3%) and RCA 33.3%. Safley and coworkers [17] reported better results in LAD and Lcx than RCA; however the success rate was higher (77%, 76% and 72%, respectively).

In our study in-hospital MACE in all CTO patients was 14.2%, which was higher than previous studies (5.1% in Olivari et al [9] and 1.9% in Rathore et al [18] study).

In-hospital MACE in our study did not differ between successful and unsuccessful PCIs; however there was a trend to higher in-hospital adverse outcome in unsuccessful group. Considering the point that procedural complications observed including tamponade while PCI of CTO were all in unsuccessful PCI emphasis on the result much more. Unlike these findings, Chen et al [19] and Hoye and coworkers7 reported higher MACE in unsuccessful group.

Several studies had reported that patients would benefit from successful recanalization, even in the absence of viable myocardium in the territory supplied by the occluded coronary artery [20].

In our study unsuccessful PCI was associated with a significantly higher rate of 12 months MACE, especially revascularization. During the 12 months following PCI of CTO, Olivari et al [9] found a significantly higher incidence of cardiac death, combined rate of cardiac death and MI, and CABG in patients with a failed procedure as compared with patients with a successful PCI. This excess of cardiac death and MI in patients with failed CTO PCI has been reported in other studies [6, 15], but it has never been observed as early as at 12 months. Unlike these studies, in the current study there was no difference according to of cardiac death and MI in follow-up period. In the other study, successful recanalization was associated with a significant reduction in subsequent CABG but not in myocardial infarction or major adverse cardiac events [21].

Successful PCI for CTO is associated with improvement in survival when compared with PCI failure in three studies [6, 7] while no survival benefit could be revealed in another one from the Mayo Clinic [5]. The current study also could not show survival benefit of successful PCI of CTO.

### Conclusion

Although in hospital outcome was the same between groups, patients with successful PCI of CTO had a better one year follow-up outcome than unsuccessful PCI. However mortality rate was the same and main complications were due to revascularization.
